# The impact of eligibility for maintenance immunotherapy on prognosis in patients with unresectable or metastatic urothelial carcinoma

**DOI:** 10.1002/bco2.119

**Published:** 2021-10-08

**Authors:** Kai Ozaki, Shingo Hatakeyama, Toshikazu Tanaka, Daisuke Noro, Noriko Tokui, Hirotaka Horiguchi, Yoshiharu Okuyama, Naoki Fujita, Teppei Okamoto, Akiko Okamoto, Yuichiro Suzuki, Hayato Yamamoto, Takahiro Yoneyama, Yasuhiro Hashimoto, Chikara Ohyama

**Affiliations:** ^1^ Department of Urology Hirosaki University Graduate School of Medicine Hirosaki Japan; ^2^ Department of Urology Aomori Prefectural Central Hospital Aomori Japan; ^3^ Department of Urology Mutsu General Hospital Mutsu Japan; ^4^ Department of Urology Odate Municipal Hospital Odate Japan; ^5^ Department of Urology Oyokyo Kidney Research Institute Hirosaki Japan; ^6^ Department of Urology Aomori City Hospital Aomori Japan; ^7^ Department of Advanced Transplant and Regenerative Medicine Hirosaki University Graduate School of Medicine Hirosaki Japan

**Keywords:** chemotherapy, maintenance immunotherapy, prognosis, radiological response, urothelial carcinoma

## Abstract

**Objectives:**

To investigate the eligibility for maintenance immunotherapy and its impact on the prognosis of advanced urothelial carcinoma treated with first‐line chemotherapy, as the selection biases of the eligible population in the JAVELIN Bladder 100 trial remain unclear.

**Methods:**

We retrospectively evaluated 213 patients (median age, 71 years) with unresectable locally advanced or metastatic urothelial carcinoma treated with platinum‐based first‐line chemotherapy between May 2003 and April 2021. The patients were categorized into the following two groups: progressive disease (PD) within four cycles (trial ineligible group) and non‐PD within four cycles (trial eligible group). The primary outcomes were the estimated proportion of trial eligible patients for maintenance immunotherapy. The secondary outcomes were the comparison of the overall survival in the trial eligible and ineligible groups and the impact of radiologic response at the second cycle on the fourth cycle.

**Results:**

Among the 213 patients, 81 (38%) were included in the trial eligible group. The trial eligible group had a significantly longer overall survival than the trial ineligible group (*P* < 0.001). Of 166 patients who had no PD within two cycles, 85 (51%) patients experienced PD within four cycles. Patients with a complete response or partial response at the second cycle had a significantly lower rate of PD at the fourth cycle (42%) than those with stable disease at the second cycle (59%, *P* = 0.031).

**Conclusion:**

We observed 38% of the trial eligible population. Overall survival was significantly different between the trial eligible and ineligible groups.

## 1 INTRODUCTION

Advanced urothelial carcinoma (UC) is a life‐threatening disease with a high mortality rate.^1^, ^2^, ^3^, ^4^ Although platinum‐based chemotherapy is the standard‐of‐care and first‐line treatment for UC, the prognosis for those patients remains poor because of chemotherapy resistance and accumulating toxicities.^5^, ^6^, ^7^, ^8^, ^9^ The JAVELIN Bladder 100 trial demonstrated that avelumab maintenance therapy is beneficial in patients with unresectable locally advanced or metastatic UC.^10^ However, this study included patients who did not present with disease progression while receiving first‐line chemotherapy (four to six cycles of gemcitabine along with cisplatin or carboplatin). To appropriately use avelumab maintenance therapy, it is imperative to identify the selection biases of the eligible population in the JAVELIN Bladder 100 trial to translate the outcomes from clinical trial to clinical practice. Therefore, we aimed to retrospectively investigate the eligibility for maintenance immunotherapy and its impact on prognosis in unresectable locally advanced or metastatic UC in real‐world practice. The goal of this study is to identify selection biases of the eligible population in the JAVELIN Bladder 100 trial.

## 2 METHODS

### 2.1 Design and ethics statement

This retrospective, multicenter study conformed to the principles of Declaration of Helsinki and was approved by the ethics committee of the Hirosaki University School of Medicine (2019–099) and all participating hospitals. Written consent was not obtained unless patients decide to be excluded (opt‐out approach).

### 2.2 Patient selection

We evaluated 218 patients with unresectable or metastatic UC (T4, N positive, or M1) who received systemic, platinum‐based, first‐line chemotherapy between May 2003 and April 2021 at one academic center and five general hospitals. We excluded five patients whose tumor response could not be evaluated (*n* = 2), who were unfit for platinum‐based regimens because of severe renal impairment (*n* = 2) or chronic pulmonary disease (*n* = 1). In total, we enrolled 213 patients in this study. From the patient records, the following variables were collected and analyzed: age, sex, Eastern Cooperative Oncology Group performance status (ECOG PS), renal function (estimated glomerular filtration rate: eGFR), clinical stage, urinary bladder or upper urinary tract UC [UTUC], and metastatic sites. Tumor stage and grade were stratified according to TNM classification (8th edition).^11^ The baseline radiological response was evaluated by RECIST version 1.1 and classified into the following four categories: complete response (CR), partial response (PR), stable disease (SD), or progressive disease (PD). Since the trial ineligible patients were not defined and considered in the trial, we defined the trial ineligible group (PD within four cycles) and trial eligible group (non‐PD within four cycles).

### 2.3 Cisplatin ineligibility

We used the modified cisplatin ineligibility criteria, considering the population difference.^12^ Cisplatin ineligibility was considered if at least one of the following criteria was met: ECOG PS > 1, creatinine clearance < 60 ml/min or eGFR < 50 ml/min/1.73 m^2^, neuropathy grade > 1, hearing loss grade > 1, and/or New York Heart Association Class III heart failure. Cisplatin ineligibility was also defined based on the following marginal criteria: ECOG PS of 1, eGFR of 50–60 ml/min/1.73 m^2^, NYHA Class II heart failure, and age >80 years. Patients with two or more marginal factors, such as ECOG PS 1 and >80 years, were considered cisplatin‐ineligible.

### 2.4 Platinum‐based chemotherapy

Chemotherapy regimens were selected according to our cisplatin eligibility guideline. Patients received one of the following drug combinations: gemcitabine plus cisplatin (GCis), gemcitabine plus carboplatin (GCarbo), GCarbo plus docetaxel (GCarboD), or MVAC (methotrexate, vinblastine, doxorubicin, and cisplatin).^4^, ^13^, ^14^, ^15^ Drug administration cycle was repeated every 28 days until disease progression or intolerable adverse events occurred. All patients were scanned by computed tomography every two cycles. If PD or intolerable adverse events occurred, the first‐line treatment regimen was discontinued and switched to second‐line treatment regimens.^16^ Given that the standard second‐line regimen was not yet defined before 2018 (pre‐pembrolizumab era), some patients were continuously treated with the first‐line therapy beyond PD. Our regimens for salvage chemotherapy included docetaxel‐based regimes (e.g., docetaxel + ifosfamide + nedaplatin or paclitaxel + gemcitabine),^17^, ^18^ or rechallenge of first‐line therapy.

### 2.5 Outcomes

The primary outcome was estimating the proportion of the trial eligible patients for maintenance immunotherapy. The secondary outcome included the comparison of the overall survival (OS) between the trial eligible and ineligible groups and the impact of radiologic response at second cycle on fourth cycle. An OS event was defined as the length of time after primary treatment to last follow‐up or any cause of death.

### 2.6 Statistical analyses

Statistical data were analyzed using BellCurve for Excel 3.2.1 (SSRI Co., Ltd., Tokyo, Japan), GraphPad Prism 7.00 (GraphPad Software, San Diego, CA, U.S.A.), and R version 4.0.2 (The R Foundation, Vienna, Austria). The difference was tested by Student's *t*‐test, Mann–Whitney *U* test, Fisher's exact test, or *χ* ^2^ test. Means with standard deviations or medians with interquartile ranges (IQRs) was used to express quantitative variables. The OS rate was estimated by log‐rank test. The effect of trial eligibility on the OS was investigated by multivariate Cox regression analyses using the inverse probability of treatment weighting (IPTW) model.^19^, ^20^ We also calculated the hazard ratio (HR) with 95% confidence interval (95% CI) after controlling the potential confounders, including patient age, sex, performance status, tumor type, TNM stage, chemotherapy types (cisplatin‐based regimen or not), subsequent use of pembrolizumab, and liver metastasis.

## 3 RESULTS

### 3.1 Baseline characteristics

The median age was 71 years (IQR: 63–78 years). Of the 213 patients, 102 (48%) had UTUC, 153 (72%) received carboplatin‐based regimens, and 70 (30%) had unresectable locally advanced (T4 or N+) disease. The median number of cycles of platinum‐based chemotherapy was 4 (IQR: 2–4) (Table 1).

**TABLE 1:** Background of patients

	All	Trial eligible group	Trial ineligible group	*P* value
***n***	213	81	132	
**Age, years (IQR)**	71 (63–78)	72 (63–78)	70 (62–77)	*0.551*
**Male, *n***	154 (72%)	60 (74%)	94 (70%)	*0.632*
**UTUC, *n***	102 (48%)	41 (51%)	61 (46%)	*0.532*
**ECOG PS > 1**	20 (9%)	3 (4%)	17 (13%)	*0.026*
**Disease status, *n***				
**unresectable locally advanced (T4 or N+)**	70 (30%)	40 (49%)	30 (23%)	*<0.001*
**metastatic (M1)**	143 (70%)	41 (51%)	102 (77%)	
**liver metastasis**	15 (7%)	3 (4%)	12 (9%)	*0.136*
**eGFR, ml/min/1.73 m** ^**2**^ **(IQR)**	57 (45–70)	57 (43–69)	57 (46–71)	*0.541*
**Local therapy (surgery or radiotherapy), *n***	34 (16%)	16 (20%)	18 (14%)	*0.377*
**First‐line regimens**				
**cisplatin‐based**	60 (28%)	21 (25%)	39 (30%)	*0.418*
**carboplatin‐based**	153 (72%)	60 (74%)	93 (70%)	
**Number of cycles of 1st‐line therapy (IQR)**	4 (2–4)	4 (4–6)	3 (2–4)	*<0.001*
**4 cycles or more, *n***	143 (67%)	81 (100%)	62 (47%)	
**Subsequent pembrolizumab therapy, *n***	60 (28%)	27 (33%)	33 (25%)	*0.189*
**Median follow up, months (IQR)**	14 (7–24)	17 (12–30)	10 (6–20)	

*Note*: IQR: interquartile range, UTUC: upper urinary tract urothelial carcinoma, ECOG PS: Eastern Cooperative Oncology Group performance status, and eGFR: estimated glomerular filtration rate.

### 3.2 Primary outcomes

**FIGURE 1:**
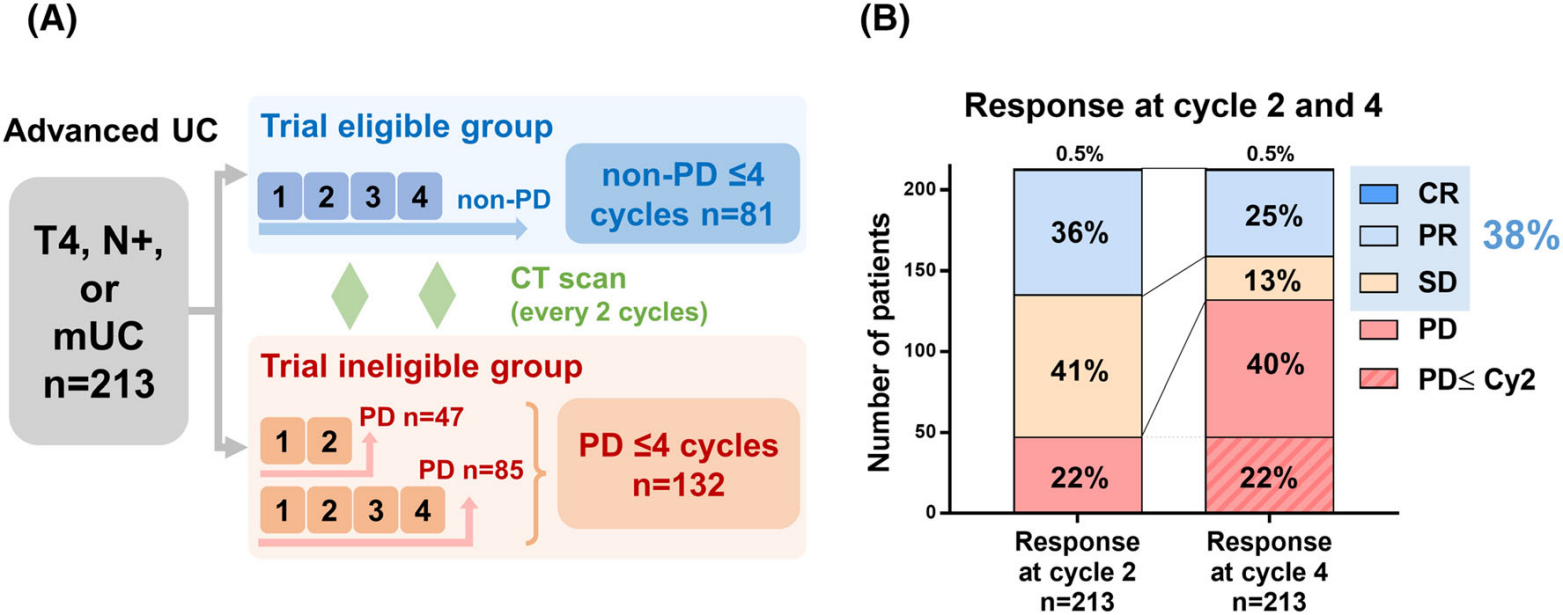
Primary outcomes. (A) Patient selection for trial eligible and ineligible groups treated with first‐line chemotherapy. (B) Proportion of trial eligible (non‐PD within four cycles) and ineligible groups (PD within four cycles). PD: progressive disease

Of 213, 132 (62%) patients had PD within four cycles of first‐line chemotherapy (trial ineligible group), suggesting that 81 (38%) patients had no PD within four cycles (trial eligible group, Figure 1A). Of 213, 47 (22%) patients had PD within two cycles of first‐line chemotherapy. Of 166 patients who had no PD within two cycles, 85 (51%) patients had PD within four cycles of first‐line chemotherapy. The change in the tumor response from second cycle to fourth cycle in CR, PR, SD, and PD were 0.5% to 0.5%, 36% to 25%, 41% to 13%, and 22% to 62%, respectively (Figure 1B). Intergroup patient baseline characteristics were significantly different in terms of the ECOG PS, metastatic disease, and number of patients with first‐line chemotherapy of four cycles or more (Table 1).

### 3.3 Secondary outcomes

**FIGURE 2:**
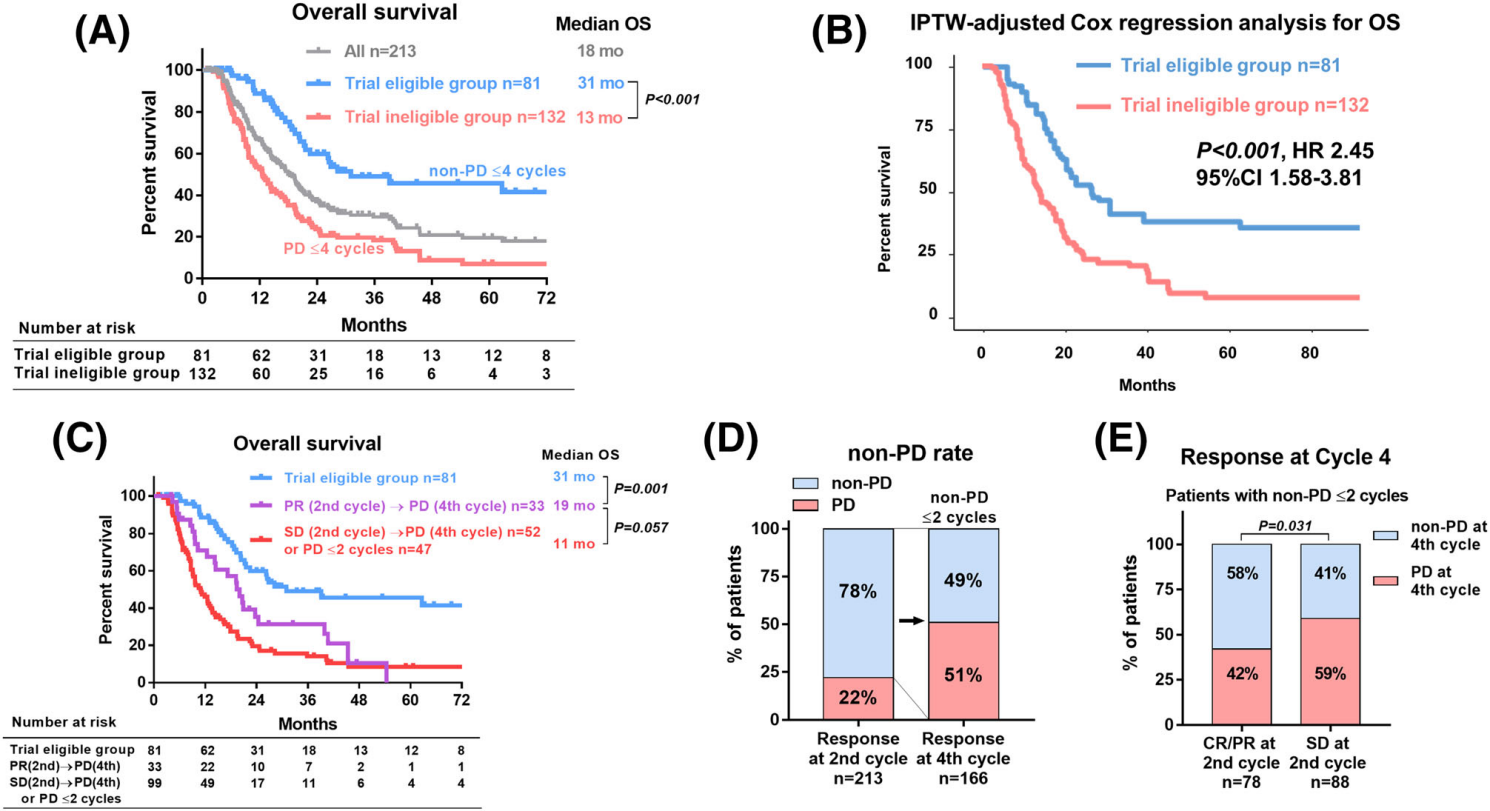
Secondary outcomes. (A) Comparison of the overall survival (OS) between the trial eligible and ineligible groups. (B) Background‐adjusted Cox regression analysis for OS. Adjusted variables were patient age, sex, Eastern Cooperative Oncology Group performance status (ECOG PS), tumor type (UTUC), TNM stage, local therapy, chemotherapy types (cisplatin‐based regimen or not), and liver metastasis. C: Comparison of OS between patients with PD within two cycles and PD at three to four cycles. D: The association of radiological response between cycles 2 and 4. CR: complete response, PR: partial response, SD: stable disease, PD: progressive disease, UTUC: upper urinary tract urothelial carcinoma. E: Tumor response at cycle 4 in patients with non‐PD at cycle 2. f: Detailed tumor response at cycle 4 in patients with non‐PD at cycle 2

The OS was significantly longer in the trial eligible group than that in the trial ineligible group (median 31 vs. 13 months, respectively, *P* < 0.001, Figure 2A). Likewise, the IPTW‐adjusted Cox regression analysis revealed that the trial ineligible group had a significantly shorter OS than the trial eligible group (*P* < 0.001; HR, 2.45; 95% CI, 1.58–3.81, Figure 2B). The OS showed no significant difference between patients with “PD ≤ 2 cycles or SD (second cycle) to PD (fourth cycle)” and those with “PR (second cycle) to PD (fourth cycle)” (median OS 11 vs. 19 months, *P* = 0.057, Figure 2C). When we evaluated the association of tumor response between second and fourth cycles, 51% of patients had PD at fourth cycle among the patients with non‐PD ≤ 2 cycles (*n* = 166, Figure 2D). In the trial eligible group, the CR/PR at second cycle was significantly associated with a lower PD rate at fourth cycle compared with SD at second cycle (42% vs. 59%, *P* = 0.031, Figure 2E). The subgroup analysis of OS in the trial ineligible group (SD at second cycles to PD at fourth cycles vs. PD within second cycles) was shown in Figure S1. The OS comparison between the trial eligible and ineligible groups in the follow‐up duration (<14 or ≥14 months) of treatment periods (before or after 2018) was shown in Figure S2. A visual abstract was in the supplement Figure S3.

## 4 DISCUSSION

In this study, we investigated the proportion of the trial eligible population for maintenance immunotherapy and the impact of trial eligibility on the prognosis of patients with unresectable or metastatic UC. Given that the JAVELIN Bladder 100 trial formed an eligible population, we need to recognize the selection biases of this population to translate the outcomes from clinical trial to practice. In our practice, less than half (38%) of patients were eligible for avelumab maintenance therapy, suggesting that more than half of patients were candidates for second‐line pembrolizumab therapy. Furthermore, the OS was significantly different between the trial eligible and ineligible groups based on the inclusion criteria. We need to know that avelumab maintenance therapy is effective in selected patients with favorable disease control. Therefore, we could not compare the outcomes of switch maintenance therapy with the other first‐ or second‐line trials in one table.^21^ In this regard, our study can provide useful information for proper understanding of treatment, although our study is not useful on deciding to perform immunotherapy or not for each patient. As the goal of this study is to identify selection biases of the eligible population in the JAVELIN Bladder 100 trial, the definition of trial ineligible group and the OS comparison does not necessarily negate the findings of JAVELIN Bladder 100 trial.

Although no study supports the effect of early administration of avelumab maintenance therapy within four cycles of first‐line chemotherapy, it might be a potential option considering an immunogenic cell death at the time of best response (i.e., after approximately two or three cycles).^22^ In our study, the OS showed marginal difference (*P* = 0.057) between patients with “PD ≤2 cycles or SD (second cycle) to PD (fourth cycle)” and those with “PR (second cycle) to PD (fourth cycle).” Patients who had an initial response to first‐line chemotherapy might have favorable effects for avelumab maintenance therapy because cytotoxic chemotherapies induce dynamic changes in the tumor immune microenvironment.^23^ However, our results showed that the OS was significantly different between the trial eligible and ineligible population, suggesting that disease progression within the first four cycles is a signal of a poor prognosis. Concerning the eligible population formed by the JAVELIN Bladder 100 trial, we need to recognize that level 1 evidence is limited in patients without PD receiving four to six cycles of first‐line chemotherapy. Nevertheless, further studies are warranted to determine the optimal timing of maintenance immunotherapy.

Predicting the tumor response on the fourth cycle from the second cycle is important for the indication of avelumab maintenance therapy. Our results showed that patients with CR/PR at two cycles had a significantly higher rate of non‐PD on the fourth cycle. Notably, 58% of patients with CR/PR at second cycle maintained CR/PR at fourth cycle (Figure 2E). However, 51% of patients without PD at second cycle experienced PD at fourth cycle. Therefore, the eligibility criteria for the avelumab maintenance therapy are narrow.

In this study, we observed a higher proportion of carboplatin‐based regimens (72%). The administration of cisplatin in all patients is not feasible because of renal impairment, older age, poor general status, and/or comorbidities,^24^, ^25^, ^26^ Nearly 60% of patients with urothelial cancer are reportedly ineligible for cisplatin‐based chemotherapies.^12^, ^27^ Although cisplatin ineligibility currently does not have any specific criteria, we used the modified cisplatin ineligibility criteria to secure a safety margin. Considering that our patients were older (median age, 71 years) than those in clinical trials (64–68 years),^5^, ^6^, ^7^, ^27^, ^28^, ^29^ obtaining a 72% proportion of patients with cisplatin ineligibility is reasonable in a clinical practice.

This study has several limitations that need to be acknowledged. The selection bias and other unmeasurable confounders were not controlled because of the retrospective study design. The statistical analysis may be underpowered because of the small sample size. Also, analyses under a heterogenous population (carboplatin‐based regimens and higher UTUC proportion) prevent generalization. Despite the limitations, this study presents the clinical implication of eligibility for maintenance immunotherapy and its impact on prognosis in unresectable or metastatic UC. However, further investigation is necessary to determine the optimal strategies of transition from the first‐line platinum‐based chemotherapy to maintenance immunotherapy.

### 4.1 Conclusion

We observed 38% of eligible patients for avelumab maintenance therapy in our practice. The OS was significantly different between the trial eligible and ineligible groups. CR or PR at two cycles was significantly associated with the eligibility for maintenance immunotherapy.

## FUNDING INFORMATION

This study was supported by Japan Society for the Promotion of Science (JSPS) Grant Numbers of 19H05556 (C.O.), 20K09517 (S.H.), 19K18603 (N.F.), 19K18575 (Y.S.), 18K16718 (D.N.), and 21K16749 (H.H.).

## CONFLICT OF INTEREST

The authors have no conflict of interest.

## AUTHORS' CONTRIBUTIONS

Kai Ozaki: Data collection.

Shingo Hatakeyama: Project development, manuscript editing, data analysis, and data collection, funding acquisition.

Toshikazu Tanaka: Data collection.

Daisuke Noro: Data collection, funding acquisition.

Noriko Tokui: Data collection.

Hirotaka Horiguchi: Data collection, funding acquisition.

Yoshiharu Okuyama: Data collection.

Naoki Fujita: Data collection, funding acquisition.

Teppei Okamoto: Data collection.

Akiko Okamoto: Data collection.

Yuichiro Suzuki: Data collection.

Hayato Yamamoto: Data collection.

Takahiro Yoneyama: Data collection.

Yasuhiro Hashimoto: Data collection.

Chikara Ohyama: Project development and critical review, funding acquisition.

## ETHICS STATEMENT

The present retrospective, multicenter study was performed in accordance with the ethical standards of the Declaration of Helsinki, and it was approved by the ethics review board of the Hirosaki University School of Medicine (authorization number: 2019–099) and all hospitals. Pursuant to the provisions of the ethics committee and the ethics guidelines in Japan, written informed consent is not required for public disclosure of study information in the case of a retrospective and/or observational study using materials, such as existing documents (opt‐out approach).

## Supporting information


**Figure S1** **Subgroup analysis of overall survival in the trial ineligible group**
Comparison of the overall survival (OS) between the “SD at second cycle to PD at fourth cycle” and “PD ≤ 2 cycles”.
Figure S2 Subgroup analysis of overall survival for the follow‐up duration and treatment periods*
Comparison of the overall survival (OS) between the trial eligible and ineligible groups in the follow‐up duration (<14 months; **A**, or ≥14 months; **B**) and treatment periods (before 2018; **C** or after 2018; **D**).
*, The approval of pembrolizumab was Dec. 2017.
Figure S3 A visual abstract
Summary of the present study
