# Supplementary material for: The impact of eligibility for maintenance immunotherapy on prognosis in patients with unresectable or metastatic urothelial carcinoma
Source: BJUI Compass. 2021 Oct 8;3(2):139–45. doi: 10.1002/bco2.119 (PMC8988805; doi:10.1002/bco2.119)
Supplement: Supplementary file 1 — Figure S1 Subgroup analysis of overall survival in the trial ineligible group Comparison of the overall survival (OS) between the “SD at second cycle to PD at fourth cycle” and “PD ≤ 2 cycles”. Figure S2 Subgroup analysis of overall survival for the follow‐up duration and treatment periods* Comparison of the overall survival (OS) between the trial eligible and ineligible groups in the follow‐up duration (<14 months; A, or ≥14 months; B) and treatment periods (before 2018; C or after 2018; D). *, The approval of pembrolizumab was Dec. 2017. Figure S3 A visual abstract Summary of the present study [file BCO2-3-139-s001.pdf]

Fig. S1

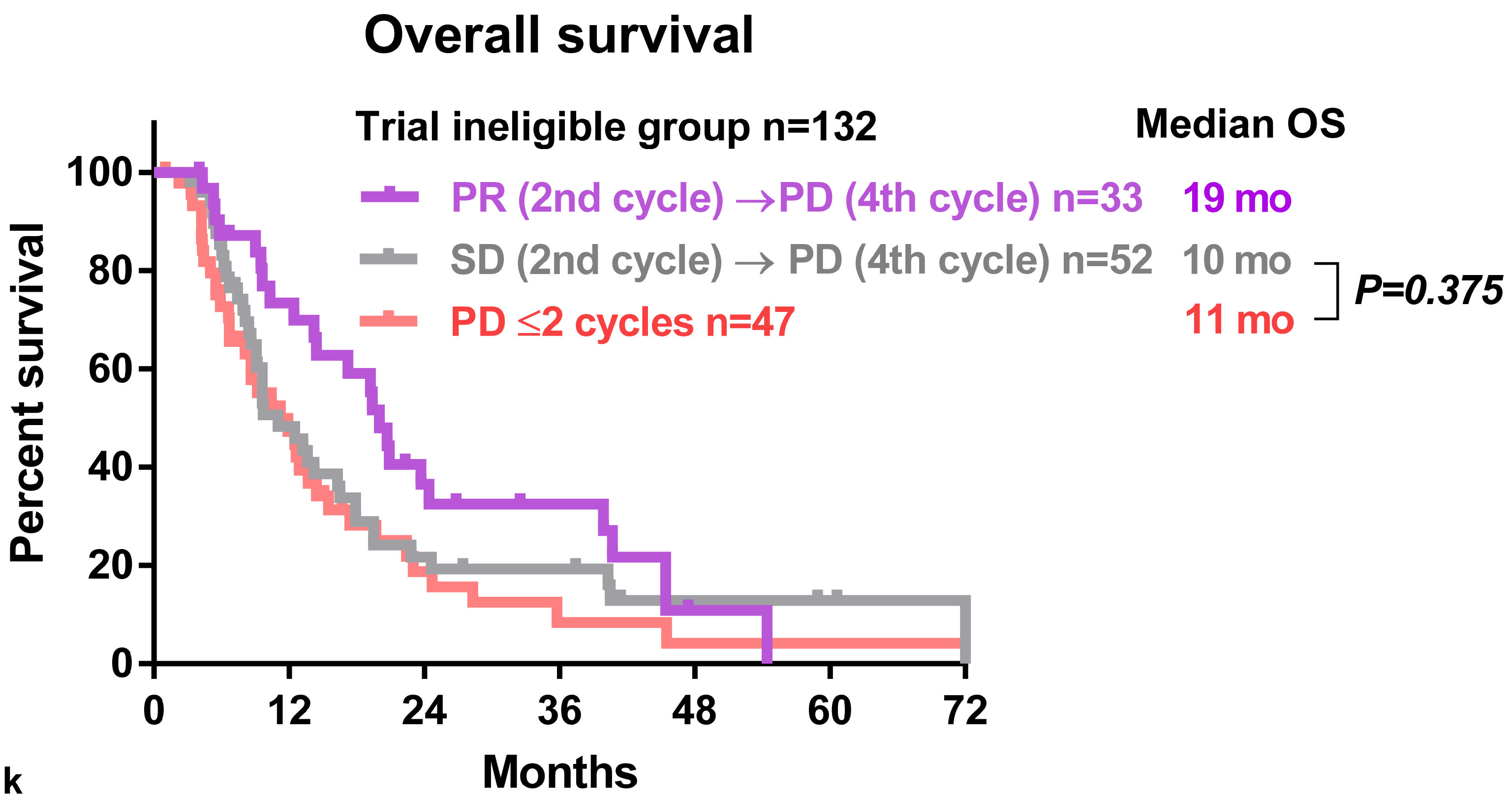

Number at risk

PR(2nd)→PD(4th)	33	22	10	7	2	1	1
SD(2nd)→PD(4th)	52	21	10	8	4	2	2
PD ≤2 cycles	47	19	7	3	2	2	2

Fig. S2

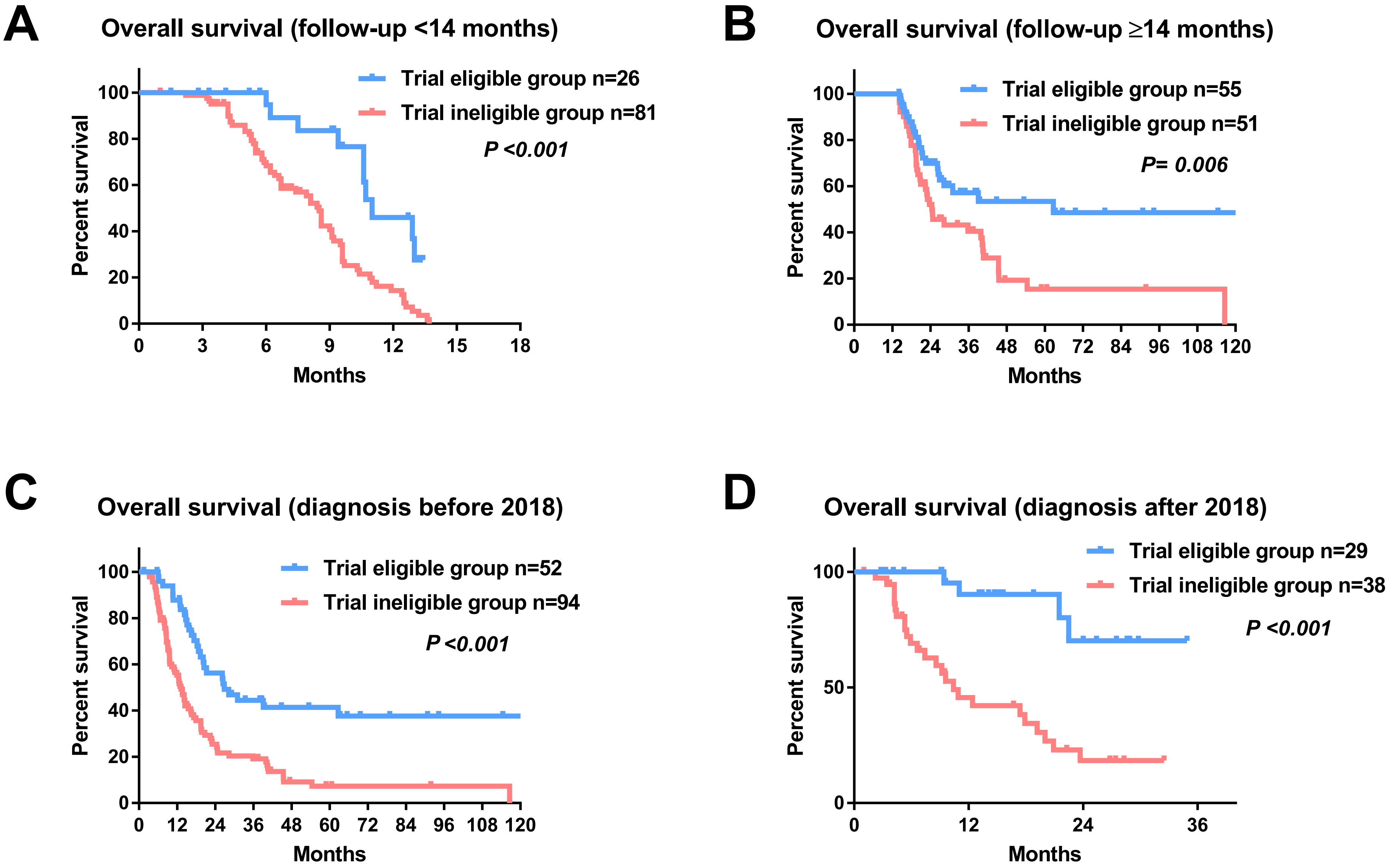

A
Overall survival (follow-up <14 months)
Trial eligible group n=26
Trial ineligible group n=81
P <0.001
Percent survival
Months
B
Overall survival (follow-up ≥14 months)
Trial eligible group n=55
Trial ineligible group n=51
P= 0.006
Percent survival
Months
C
Overall survival (diagnosis before 2018)
Trial eligible group n=52
Trial ineligible group n=94
P <0.001
Percent survival
Months
D
Overall survival (diagnosis after 2018)
Trial eligible group n=29
Trial ineligible group n=38
P <0.001
Percent survival
Months

The impact of eligibility for maintenance immunotherapy on prognosis in patients with advanced UC

Fig. S3 Visual abstract

Methods

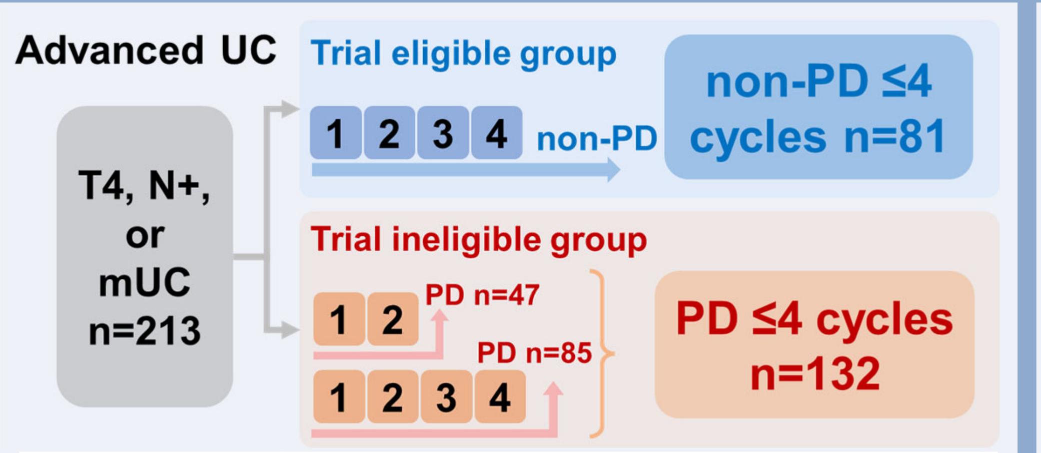

Primary	The estimated proportion of trial eligible patients for maintenance immunotherapy
Secondary	The comparison of the OS in the trial eligible and ineligible groups

Outcomes

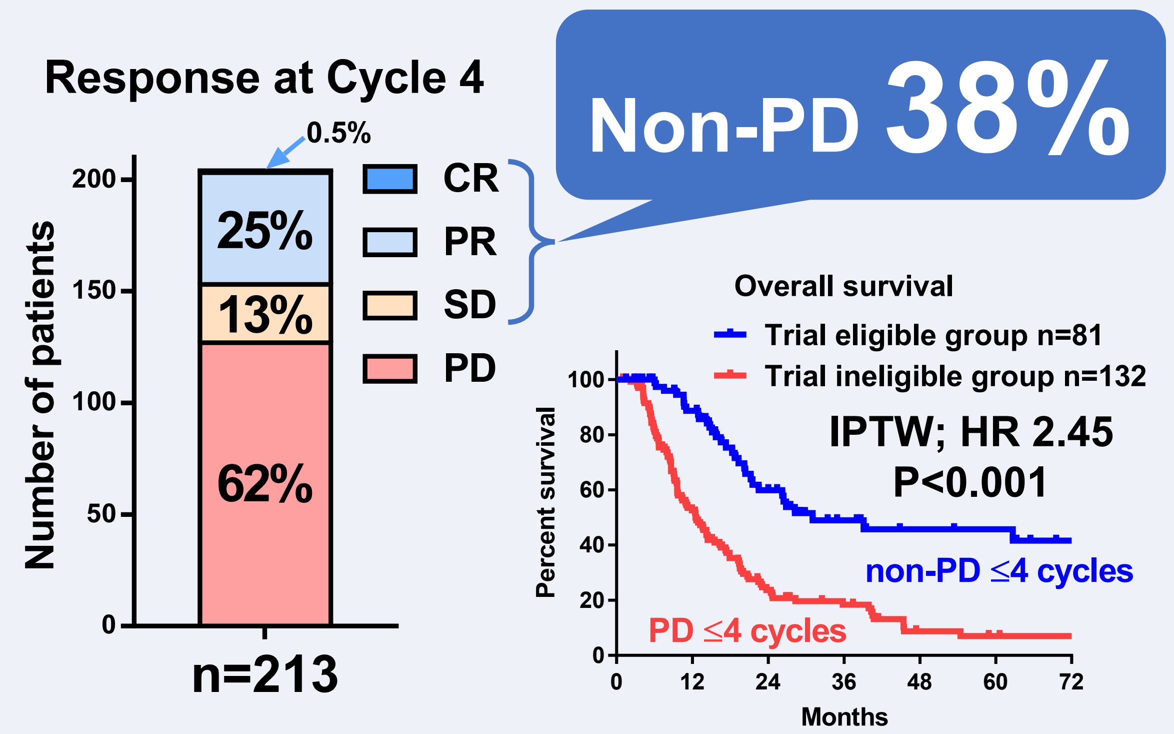

The trial eligible population was estimated at 38%. A significantly longer OS was observed in the trial eligible group.

Ozaki K., et al
